# Meningovascular Syphilis: A Case of a Young Man Presenting With Acute Stroke and Pulmonary Emboli

**DOI:** 10.7759/cureus.44568

**Published:** 2023-09-02

**Authors:** Alexa Ragusa, Adrian Kapustka, Latha Ganti, Shayne Gue

**Affiliations:** 1 Emergency Medicine, University of Central Florida (UCF)Hospital Corporation of America (HCA) Florida Healthcare Graduate Medical Education (GME), Kissimmee, USA; 2 Emergency Medicine, University of Central Florida College of Medicine, Orlando, USA

**Keywords:** pulmonary embolism, hiv, stroke, neurosyphilis, meningovascular syphilis, syphilis

## Abstract

Syphilis is caused by the spirochete *Treponema pallidum* and classically progresses through a series of stages with increasing symptomatology if unrecognized and untreated. Importantly, central nervous system invasion can occur at any stage, which can lead to variable presentations of neurosyphilis. One such manifestation is meningovascular syphilis, which causes thrombosis of the cerebral vasculature, leading to stroke-like symptoms such as hemiplegia and aphasia. Young, healthy patients may present with these symptoms without any risk factors typically associated with the pathophysiology of cerebrovascular accidents. Further, patients living with HIV who present with stroke-like symptoms should have an even higher suspicion for neurosyphilis as a potential diagnosis. We present a case report of a 31-year-old male with sudden left-sided weakness and numbness who tested positive for both *Treponema pallidum* and human immunodeficiency virus (HIV).

## Introduction

Syphilis is a sexually transmitted infection caused by the *Treponema pallidum* spirochete, which can spread to the central nervous system in a matter of days following exposure. This potential early spread may result in neurologic manifestations occurring at any stage of the infection, not only in tertiary syphilis [1]. Primary syphilis classically presents with a chancre (a single, nontender genital ulcer) within 9-90 days after infection [2]. During this stage, the chancre may go unnoticed, leading to the non-treatment of the infection in its primary stage. When primary syphilis goes untreated, the infection can progress to secondary syphilis, which typically occurs within 12 weeks (but may take up to 12 months following the initial manifestations). This stage is marked by more widespread symptomatology. Classically, a rash presents on the trunk, hands, or feet and may include condyloma lata around the anogenital region in some patients [2]. Further lack of diagnosis and/or treatment will eventually lead to the progression to tertiary syphilis. This stage may involve a spectrum of clinical manifestations, including gummatous syphilis, cardiovascular syphilis, or late neurosyphilis [2]. As described, untreated syphilis may progress to neurosyphilis at any stage, presenting as acute meningeal syphilis, meningovascular syphilis, paretic neurosyphilis, tabetic neurosyphilis, or may remain asymptomatic [3]. Diagnosis is typically obtained by first utilizing non-specific lipoidal tests such as rapid plasma regain (RPR) and the Venereal Disease Research Laboratory (VDRL) for screening, followed by treponemal-specific tests used to confirm positive lipoidal results [1]. The standard treatment for neurosyphilis is aqueous crystalline penicillin G, administered as 3-4 million units IV every 4 hours or as a continuous infusion of 18-24 million units per day for 10-14 days [4]. In recent years, rates of syphilis have increased, with some sources citing as much as a 700% increase amongst men in the US from 2011-2021 [5]. Therefore, rates of neurosyphilis have likely also increased, although there are limitations to obtaining accurate epidemiological data given inconsistencies in reporting. We present a unique case of a young patient with an acute stroke who later tested positive for syphilis and HIV.

## Case presentation

A previously healthy 31-year-old male presented to the emergency department following a one-day history of left-sided weakness and numbness. The night prior, the patient experienced a sudden onset of severe fatigue along with left-sided numbness. He reported having similar episodes of fatigue over the past few months without any focal neurologic symptoms in prior episodes. On the day of admission, the patient began experiencing left-sided weakness and left facial droop. The patient also reported a gradual onset of intermittent, sharp left-sided chest pain for the past week, which resolved after taking aspirin. He denied any radiation of the pain or recognized aggravating factors. Further, he denied dyspnea, near syncope, edema, abdominal pain, fever/chills, or other systemic symptoms. Past medical history was unremarkable, with no diagnosed medical conditions, recent illnesses, prior hospitalizations or surgeries, trauma, or illicit drug use. He did report previous sexual intercourse with men but denied any sexual activity in the last seven years and had never been previously diagnosed with a sexually transmitted infection. He also reported tobacco cessation approximately eight months before admission.

**Figure 1:**
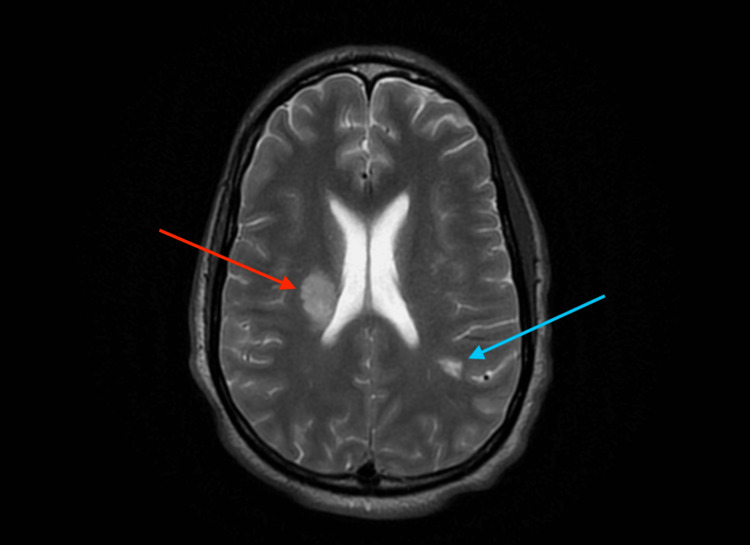
MRI brain demonstrating right periventricular, middle cerebral artery territory acute ischemic stroke (red arrow) with a left enhancing parietal lobe lesion (blue arrow).

A physical examination revealed mild tachycardia but otherwise unremarkable vital signs. A complete neurologic exam and the National Institute of Health Stroke Scale (NIHSS) were recorded on emergency department arrival. The patient was alert and oriented to person, place, and time, with normal speech, normal memory, and an intact thought process. Pupils were equal, round, reactive to light and accommodation, and extraocular movements were intact without ptosis; however, the exam was significant for the patient who had no blink to threaten his peripheral vision bilaterally. Facial sensation was intact bilaterally. Facial muscle strength was normal and equal bilaterally. Hearing was normal bilaterally. The shoulder shrug was strong and equal bilaterally. The tongue protruded midline and moved symmetrically. Sensation to both pain and light touch and two-point discrimination were intact in all four extremities. On motor examination, pronator drift was observed in the left upper extremity, as well as drift in the left lower extremity. Coordination testing was abnormal, with finger-to-nose testing revealing ataxia in the left upper extremity; however, heel-to-shin testing was normal bilaterally, Romberg was negative, and rapid alternating movements were normal. Th gait was steady with a normal base. His initial NIHSS was 3 for weakness and drift of the left upper and lower extremities as well as limb ataxia in the left upper extremity. A cardiac exam revealed a normal appearance of the chest without lifts, heaves, or thrills. The heart rhythm was normal, with mild tachycardia. There were no murmurs, gallops, or rubs auscultated. S1 and S2 were heard and were of normal intensity. No edema was noted in the bilateral lower extremities. The remainder of his physical exam was unremarkable, including documentation of a skin examination revealing no rashes or lesions. An electrocardiogram (ECG) in the emergency department revealed sinus tachycardia and no other abnormalities. Non-contrast computed tomography (CT) of the head was interpreted by radiology and summarized with the impression of "a small hyperdensity in the high left parietal lobe, possibly representing a small subarachnoid hemorrhage or hyperdense lesion." Computed tomography angiographies (CTAs) of the head and neck were negative for large vessel occlusion, aneurysms, or hemodynamically significant stenosis. However, the chest was partially visible on the CTA of the neck, which revealed bilateral pulmonary emboli. A static magnetic resonance imaging (MRI) of the brain was ordered to rule out intracranial hemorrhage before starting anticoagulation for the pulmonary emboli. MRI of the brain revealed multiple signal abnormalities, potentially due to active demyelinating plaques or acute infarcts (Figure 1). Neurology interpreted this study as "right periventricular, middle cerebral artery territory acute ischemic stroke with a left enhancing parietal lobe lesion." After initial imaging results had been reported, the patient was sent for a CTA of the chest, which revealed extensive bilateral pulmonary emboli, including a saddle embolus in the distal left pulmonary artery.

At this point in the assessment, numerous diagnoses were considered, including septic emboli, other cardioembolic source, undiagnosed patent foramen ovale, underlying clotting disorder, multiple sclerosis, or acute disseminated encephalomyelitis. Laboratory analysis was significant only for anemia with a hemoglobin of 10.3 g/dl, mildly prolonged PT/INR of 1.2, and an elevated D-dimer of 96,172 mg/L. White blood cell count (and differential), electrolytes, renal function, hepatic function tests, and troponins were within normal limits. The urine drug screen was negative. Although the patient met only one criteria for SIRS (mild tachycardia) and his qSOFA score was 0, he lacked other risk factors for stroke and pulmonary embolism. Therefore, an infectious etiology remained high on the differential, so blood cultures were drawn, and the patient was started on vancomycin and piperacillin/tazobactam. Neurology and critical care teams were consulted. The decision was made to perform a lumbar puncture followed by a heparin infusion to treat the pulmonary emboli. The patient was admitted to the intensive care unit (ICU) for close observation and further evaluation and management.

In the ICU, a lumbar puncture was performed by the neurology team following the administration of empiric antibiotic therapy (which may have impacted the results). Analysis of CSF revealed a WBC of 4/mm3, total protein of 78 mg/dl, and glucose of 50 mg/dl. Opening pressures were not documented. Duplex ultrasonography of the bilateral lower extremities was negative for deep vein thrombosis. Cardiology was consulted, and an echocardiogram was completed, which did not show any right ventricle strain pattern as a result of the pulmonary emboli. The bubble study was negative, ruling out a potential patent foramen ovale or another septal defect. A transesophageal echocardiogram (TEE) revealed no embolic phenomenon and no evidence of a left atrial appendage thrombus or any other intracardiac thrombus, ruling out endocarditis or another cardioembolic source as a cause for the ischemic stroke.

During his hospital stay, the patient began to experience spiking fevers intermittently at night. Previously drawn blood cultures were negative for growth over five days. The patient tested positive for HIV with a CD4 count of 21 cell/mcL. At this point, infectious disease was consulted, and he was started on a one-pill regimen of bictegravir, emtricitabine, and tenofovir alafenamide, as well as oral trimethoprim-sulfamethoxazole (800mg-160mg) for PCP prophylaxis. Given the new diagnosis of HIV, additional infectious disease testing was ordered. The patient also tested positive for syphilis and was started on intravenous penicillin G (4 million units every 4 hours for 14 days). He was discharged 11 days later to inpatient rehab.

Unfortunately, seven days after hospital discharge, while in inpatient rehab, the patient had acute worsening of his left-sided weakness and new aphasia and was therefore re-evaluated by neurology and re-admitted to the hospital. He then began displaying signs of meningitis, including fevers and nuchal rigidity. He also reported vision changes in the right eye, described as progressively worsening blindness, and retinal hemorrhages were discovered on fundoscopy. A second lumbar puncture was performed, which resulted for cytomegalovirus (CMV) and varicella zoster virus PCR. The patient was then started on intravenous ganciclovir for CMV retinitis. The patient was discharged from our facility on hospital day 22 and was transferred to a nearby tertiary care facility for further care by a retina-specialized ophthalmologist.

## Discussion

Meningovascular syphilis is one clinical manifestation of neurosyphilis that may cause several sequelae of the disease not typically seen in a young patient population. Although not commonly recognized or reported, progression to meningovascular syphilis is estimated to occur in 3.2%-15% of syphilis cases (assuming no therapeutic intervention) [1]. Meningovascular syphilis is caused by an endarteritis of vessels throughout the central nervous system (CNS), resulting in thrombosis and infarction. Symptoms are site-specific and commonly involve the middle cerebral artery or its branches. Manifestations include contralateral hemiplegia, hemianesthesia, homonymous hemianopsia, and aphasia [1]. Typically, these clinical features are rarely expected to occur in young individuals with no history of cerebrovascular risk factors (high blood pressure, diabetes, hypercholesterolemia, and smoking), making the specificity of a clinical diagnosis higher in this population [1]. While the causal syphilis infection can be treated, the neurologic effects of meningovascular syphilis are often irreversible. The goal of treatment is to halt the progression of these neurologic sequelae rather than reverse the effects of meningovascular syphilis.

Our patient presented with a right periventricular MCA, territory acute ischemic stroke, and symptoms of left-sided weakness and numbness. His only risk factor for stroke was a history of smoking, with cessation approximately eight months before the emergency department visit. Krishnan et al. [6] report a similar case where a 39-year-old man presented with acute global aphasia and right-sided facial weakness with no conventional cardiovascular risk factors and was diagnosed with neurosyphilis. Another case involved a 30-year-old man presenting with sudden-onset speech disturbance, right-sided weakness, and mild right facial weakness who was discovered to test positive for syphilis as well [7]. These cases signify the need to evaluate for syphilis when young patients with few, if any, cerebrovascular risk factors present with symptoms of stroke.

HIV and syphilis coinfection has also been on an exponential rise in recent decades [8]. In the 1990s, the prevalence of syphilis decreased in many regions, reaching a historic low between 2000-2001 (likely due to changes in sexual behavior surrounding the HIV epidemic) [8,9]. However, since 2001, the rates of primary and secondary syphilis have been rising each year, increasing 28.6% during 2020-2021 [9]. Syphilis infection may lead to host immunosuppression and weaken the response to HIV, thus increasing the chances that exposure may lead to infection with HIV [10]. Increasing rates of coinfection, along with a synergistic mechanism of acquisition, warrant a potential need for dual testing when a patient tests positive for either pathogen.

Another interesting finding in our patient was the presence of numerous pulmonary emboli discovered on imaging. The patient had been complaining of left-sided chest pain for the past week, which seemed to resolve after he took aspirin. After the incidental finding on the CTA of the neck, a CTA of the chest revealed several emboli throughout the pulmonary vasculature. Although patients living with HIV are known to be at higher risk for venous thrombosis, there is little evidence suggesting a correlation between syphilis infection and pulmonary embolism. A recent case report discussed the death of a 40-year-old man in a psychiatric hospital, where an autopsy revealed pulmonary embolism as the cause of death and subsequent evidence of neurosyphilis on examination of the brain [11]. This potential correlation, also found in our patient, may be worthwhile to further investigate, given the broad spectrum of illness presentations seen in patients with syphilis.

## Conclusions

This case highlights a unique presentation of a 31-year-old man presenting with an ischemic stroke of the right MCA and pulmonary emboli, who was subsequently diagnosed with syphilis and HIV. The patient’s initial presentation of CVA was atypical for a young patient whose only risk factor was a history of smoking. Neurosyphilis is often overlooked, given other far more common etiologies that contribute to cerebrovascular accidents. With an increased prevalence of syphilis coinfection in HIV-positive individuals, patients living with HIV presenting with stroke symptoms should have neurosyphilis as a differential consideration.
